# Multistructure-Based Collaborative Online Distillation

**DOI:** 10.3390/e21040357

**Published:** 2019-04-02

**Authors:** Liang Gao, Xu Lan, Haibo Mi, Dawei Feng, Kele Xu, Yuxing Peng

**Affiliations:** 1 National Key Laboratory of Parallel and Distributed Processing, College of Computer, National University of Defense Technology, Changsha 410073, China; 2 School of Electronic Engineering and Computer Science, Queen Mary University of London, London E14NS, UK

**Keywords:** deep learning, knowledge distillation, distributed architecture, supplementary information

## Abstract

Recently, deep learning has achieved state-of-the-art performance in more aspects than traditional shallow architecture-based machine-learning methods. However, in order to achieve higher accuracy, it is usually necessary to extend the network depth or ensemble the results of different neural networks. Increasing network depth or ensembling different networks increases the demand for memory resources and computing resources. This leads to difficulties in deploying depth-learning models in resource-constrained scenarios such as drones, mobile phones, and autonomous driving. Improving network performance without expanding the network scale has become a hot topic for research. In this paper, we propose a cross-architecture online-distillation approach to solve this problem by transmitting supplementary information on different networks. We use the ensemble method to aggregate networks of different structures, thus forming better teachers than traditional distillation methods. In addition, discontinuous distillation with progressively enhanced constraints is used to replace fixed distillation in order to reduce loss of information diversity in the distillation process. Our training method improves the distillation effect and achieves strong network-performance improvement. We used some popular models to validate the results. On the CIFAR100 dataset, AlexNet’s accuracy was improved by 5.94%, VGG by 2.88%, ResNet by 5.07%, and DenseNet by 1.28%. Extensive experiments were conducted to demonstrate the effectiveness of the proposed method. On the CIFAR10, CIFAR100, and ImageNet datasets, we observed significant improvements over traditional knowledge distillation.

## 1. Introduction

The development of deep learning [1,2] has led to a leap in the fields of computer vision [3,4,5,6] and natural language processing [7,8,9,10,11]. In image recognition in particular [4,12,13], recognition accuracy has reached a high level by using deep-learning methods. However, high-quality models are often accompanied by huge parameter quantities, huge computing resources, and huge storage requirements [3,14,15]. The huge demand for resources is an important obstacle to the promotion and use of deep-learning models in the industry. Especially in resource-preferred scenarios such as FPGA, mobile devices, and microcomputers, the contradiction between performance improvement and resource occupancy is more intense. Traditionally, training a deeper network or merging multiple models [16,17] may achieve better performance, but this cannot avoid the growth of resource consumption. The problem of how to improve performance without increasing network size has received extensive attention. Some training methods, such as model compression [18,19] and model pruning [20], have been proposed to solve this problem.

The distillation [21,22] method (that is, teacher–student training) is effective to solve the contradiction between network scale and network accuracy. Distillation is mainly used for classification problems. Ground-truth labels in supervised learning problems are usually of the one-hot type, only focused on the truth category. The basic idea of distillation is to extract the subcategory information of the network. Although forcing the classification of the sample to the ground-truth label is effective, it is not necessarily optimal, as it ignores the similarity information of samples in categories. Learning a similarity matrix [23,24] can preserve the similarity between classes, but sample characteristics are neglected. Category-similarity information for different samples is different. In knowledge distillation, a complex large network called ‘teacher’ extracts sample-level category-similarity knowledge (sample classification probability *P*); then, students (simple network) use it as their training targets. The teacher calculates class probability *P* for each sample and uses it to guide students’ learning. Knowledge distillation reduces the difficulty of students’ optimization. The student network that cannot learn complete interclass similarity due to its structural constraints would be better optimized by learning from the teacher. Compared with the ground-truth label, class probability contains the samples’ similarity knowledge in all categories, which enhances the effect of students’ learning.

Traditional knowledge is static and two-stage, which enhances the performance of the student network, but it is only useful for small networks that perform poorly. However, the performance improvement of large networks is more meaningful and difficult. The deep mutual-learning (DML) method [25] uses a group of students to improve their performance by learning from each other. Students continuously learn from other’s classified probability so that each student can maintain the same class probability as others. Every student is better than traditional supervised learning. In DML, several large networks are promoted from each other, but there are still shortcomings: continuous mutual imitation weakens the generation of complementary information so that it reduces the final generalization ability, the number of students is limited by the single machine’s resources, and it is difficult to achieve effective expansion.

In this paper, we propose a multistructural model online-distillation method. Compared with other work, our method not only has stronger compatibility (multistructure), scalability (distributed environment), but also better performance (higher-precision improvement). Our method is mainly based on two premises: a stronger teacher educates better students, and appropriate distillation methods can reduce information loss.

Better teachers are made up of differentiated students. In knowledge distillation, a good teacher determines the upper limit of the student model. We adopted three strategies to strengthen the teacher model. We trained a group of students under distributed conditions. Using the weighed average ensemble method [16,17], we summarized student models’ information to form a teacher model. The ensemble effect is mainly influenced by the complementary knowledge between the student models. We extended the distillation method to a distributed framework so that we could accommodate more student models to help the teacher reduce the risk of overadaptation. In addition, students of different structures have more complementary information; we used soft labels as the information-exchange medium to jointly train networks of different structures.

Gradually intensifying knowledge distillation reduce information loss. For continuous mutual imitation, there is a risk of information consistency. We used interval distillation to increase the information diversity of the student model by inserting independent training. Although the use of interval distillation enhances overall information growth, it faces loss-function switching, and sudden changes may result in the loss of network information. We gradually increased teacher constraints so that students’ loss became flat to avoid information loss. These methods reinforce the feedback between teachers and students, and increase information complementarity. Our results go beyond the previous distillation method.

The shortcoming of our method is that multiple student nodes lead to greater training overhead and greater information redundancy. Fortunately, there was no change in time complexity and space complexity when used, and better performance than previous methods was achieved. In general, multistructure online distillation enhances information diversity in the distillation process and improves the accuracy of the model through multinetwork cooperation. Extensive experimentation was carried out on image-recognition issues using popular network architectures and achieved the highest performance improvement. Our approach has the following advantages:

1. Effectively utilizes diversity between different structural models.
2. Demand for network resources is low and applicability is stronger.
3. Network performance achieved the highest improvement while not increasing resource occupation.

The organization of this paper is as follows. Section 2 briefly reviews the related work, and Section 3 describes our approach. The experiment is shown in Section 4, and we summarize this work in Section 5.

## 2. Related Work

There is a lot of work to improve the performance of deep-learning models through knowledge transfer. This section describes some of the network-interaction methods that are relevant to our work.

In the study of network interaction, some distributed methods [26,27,28,29,30] are used to accelerate the training of the network. For example, the parameter average MA [28,29,30] method and the distributed stochastic gradient descent algorithm [31] are widely used to accelerate the training process through multinode information exchange. In addition, multitasking learning [32,33,34,35] mainly plays a role in feature selection to lift accuracy. In multitask learning, there are multiple training objectives at the same time. The network has different characteristics for feature selection based on different targets, forcing the network to learn complementary features to improve network performance. The ensemble method integrates the output of multiple networks to generate more flat predictions than a single network, improving the generalization of the model. There are many studies integrating multiple models, such as separated score integration (SSI) [36], Bayesian model averaging [37], and score fusion based on alpha integration [38]. These methods inspired us to design our own training framework.

Our approach is based on knowledge distillation [21,22,25,39,40]. Hinton, Geoffrey proposed knowledge distillation [22] that allows small models to learn the knowledge in a pretrained large model. The main motivation for distillation is that the teacher model’s soft label provides knowledge of interclass similarity that cannot be provided by the ground-truth goal. The traditional method of knowledge distillation is limited by the direction of information flow, so it cannot improve performance on large networks. Lan, Zhu, and Gong improved knowledge distillation and proposed distillation method ONE [41], which uses a set of multibranch student models to learn from each other. Mutual imitation to enhance the learning of the target network, but ONE shares the low-level features lacking diversified feature learning. Codistilling [39] online distillation through large-scale distributed training can accommodate more nodes for training and accelerates the training process.

Existing distillation methods lack the inclusiveness of the network structure, which limits the complementary information that the structure can provide. In addition, we found that the distillation process forced multiple models to fully adapt to uniform soft-label output, which actually weakened the diversity of student development, which was detrimental to the end result.

In this paper, we created a distributed cross-structure online-distillation method and loosened distillation constraints to enhance network diversity. We used soft labels that are independent of the network structure as the information-exchange medium, abandoning the parameters and gradients commonly used for traditional distributed-information exchange. Soft labels meet the needs of information exchange during the distillation process and are structurally independent, allowing our students to combine multiple structural models. The ensemble method is helpful to form teachers who are better than previous knowledge-distillation methods. Considering that continuity distillation leads to the simplification of model information, we used interval distillation to enhance model diversity, and gradually enhanced distillation to capture small information differences.

## 3. Methodology

This section introduces our methods. First, in the overview, we introduce our methods and principles. Then, details of the implementation of the method are introduced in the following section.

### 3.1. Overview

**Figure 1:**
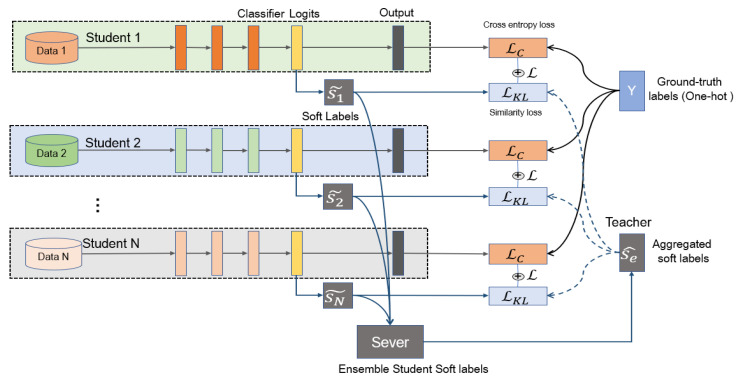
Overview of our approach. Multiple students work together, each student is a separate model, and the teacher was the aggregated information (${\hat{S}}_{e}$) from multiple student networks. First, students train their models by minimizing cross-entropy loss $L_{C}$ to learn from ground-truth label *Y*. Then, the server aggregates student information (${\tilde{S}}_{1},{\tilde{S}}_{2},\ldots ,{\tilde{S}}_{N}$) to generate teacher information (${\hat{S}}_{e}$). Finally, the teacher feeds back the student by minimizing Kullback–Leibler divergence $L_{KL}$ between fixed aggregated soft labels ${\hat{S}}_{e}$ and student soft labels ${\tilde{S}}_{i}$ during the distillation phase.

As Figure 1 shows, we used multiple networks for distributed joint training; each network is a student. The teacher network is the knowledge (${\hat{S}}_{e}$) aggregated by all student networks. Training is divided into two stages, independent training and distillation. In the independent-training phase, the model learns the relationship between training data and the ground-truth label by minimizing cross-entropy loss. After a certain independent-training period, students’ soft labels are uploaded to the server. In the server, we aggregated student information by calculating the weighted average values of their soft labels (${\tilde{S}}_{1},{\tilde{S}}_{2},\ldots ,{\tilde{S}}_{N}$). Then, the aggregated soft labels (${\hat{S}}_{e}$) were sent to each student to guide students’ distillation training. Then, in the distillation process students are optimized by minimizing Kullback–Leibler divergence between fixed aggregated soft labels ${\hat{S}}_{e}$ and students’ soft labels ${\tilde{S}}_{i}$. After distillation is finished, we return to the independent-training stage.

**Distributed Training.** We distributed training a group of students and, by regularly aggregating information in the server, we used the aggregated information as the teacher in distillation. Soft labels were used for information transmission instead of parameters or gradients. Soft labels have network-independent characteristics, so we could mix networks of different structures. Soft labels do not need to be updated at every step, longer transmission interval is allowed. Our distributed training has three advantages: reducing network overhead, compatibility with different network structures, and expansion-capacity enhancement.

**Figure 2:**
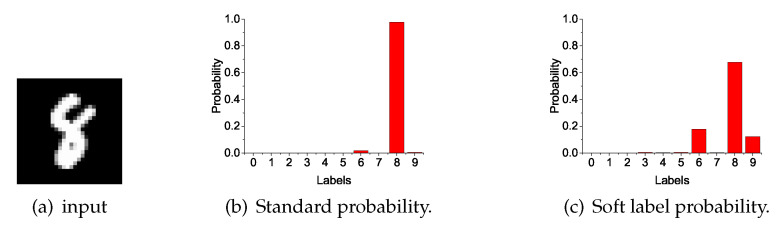
On the MNIST dataset, Alexnet after 50 rounds of training outputs. (**a**) One sample from MNIST handwritten digit database, (**b**) standard probability (temperature = 1) and (**c**) soft-label probability (temperature = 3) for input. Soft labels can express the class-similarity relation more comprehensively.

**Ensemble gets better teachers.** The distillation method improves network performance because of supplementary information. Different network structures and different initializations can generate different knowledge. The model expresses the relationship between data and labels by probability output. Soft labels (as shown in Figure 2) are the softening probabilities that not only concern sample classification, but also sample similarity. The similarity relationships provide additional information during distillation. We collected soft labels from all students and ensembled them by calculating the weighted average values. The aggregated soft labels had better generalization performance, abstracted as the teacher in distillation.

**Better distillation.** Additional knowledge is important for distillation. Compared with the previous method, we used interval distillation to expand information diversity. In our approach, the teacher is generated through the aggregation of students. The teacher has more supplementary information, while the students are more diverse. The paradox is that all students learn from the same teacher, which would make students’ knowledge consistent and lack complementarity. We joined an independent-training phase without distillation constraints, so that students could learn independently and have diversified development. Switching the loss function between independent training and distillation causes the gradient value to change dramatically, which may lead to loss of model information. We gradually increased the limitation of teacher guidance (as Equation (10) shows) in the distillation process to retain more details.

**Figure 3:**
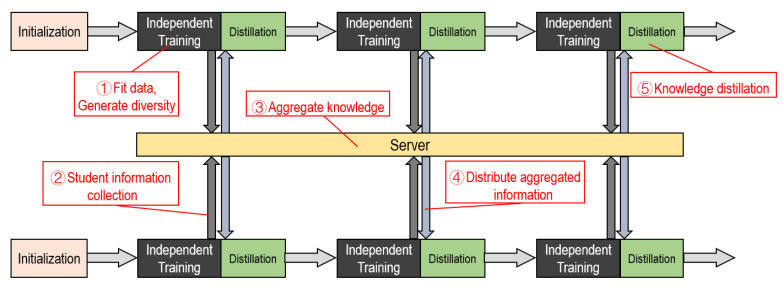
Our method’s training process.

The training process is shown in Figure 3. Our approach is a multicycle process. Each cycle can be roughly divided into five steps. These are independent training, distillation training, and information, transmission, reception, aggregation.

In this paper, we propose a cross-architecture online-distillation approach that improves classification accuracy without changing the network structure. We combined distillation and independent training in a cycle. The weighted average ensemble method was used to synthesize teacher knowledge; information on multistudents was extracted and utilized. The teacher guides students training through knowledge distillation. Student performance improved through three stages: learning from local data distribution, generating teacher information by aggregating students’ information, and learning from the teacher by distillation.

### 3.2. Independent Training

For a *C* class-classification task, assume there are *N* samples of input $X=\{x_{i}|i\in \left(1,2,\ldots ,N\right)\}$, and the corresponding ground-truth labels $Y=\{y_{i}|i\in \left(1,2,\ldots ,N\right)\}$. In general, ground-truth label $y_{i}$ is a one-hot vector with a dimension of 1×C, $y_{i}$ is equal to 1 only in the position of the category to which sample $x_{i}$ belongs, and equal to 0 in other positions. We take the *i*-th student as an example to introduce the training steps.

The student networks optimize the model by minimizing cross-entropy loss between predicted value $p_{i}$ and ground-truth label $y_{i}$ during the independent-training process. Students do not have information interaction, which is conducive to increasing the diversity of model information. Students’ loss function could be freely chosen according to their respective situations. Here, we take the cross-entropy-loss function as an example:

(1) $$L_{C}\left(Y|P\right)=-\frac{1}{N}\sum_{i=0}^{N-1}\sum_{k=0}^{C-1}y_{i}^{k}*logp_{i}^{k}$$

where $p_{i}^{k}$ represents the normalized probability that a student model $F_{s}$ classifies input $x_{i}$ as the *k*-th class.

Logits is the output of the penultimate layer of the convolutional neural networks (CNN) used to classify the problem. In the CNN, input $x_{i}$ is subjected to feature processing to obtain a feature vector $f(x_{i})$ (assuming the $f(x_{i})$ dimension is 1×m). Then, we used feature-classification layers (linear layer) to map the feature space to the sample mark space by linear transformation, and the output is logits. The linear relationship between logits $g_{i}$ and feature $f(x_{i})$ can be abstracted as $g_{i}=f\left(x_{i}\right)*w+b$ (where dimension of *w* is m×C, *C* is the number of classes), and logits $g_{i}$ is a vector of dimension 1×C. In general, logits $g_{i}$ is normalized by the softmax layer to obtain classification-probability output $p_{i}$. Assume that logits were obtained by inputting $x_{i}$ of the student network $F_{s}$ is $\left[g_{i}^{1},g_{i}^{2},\ldots ,g_{i}^{C}\right]$. Use the following formula to calculate normalized classification probability $p_{i}^{k}$.

(2) $$p_{i}^{k}=\frac{exp(g_{i}^{k})}{\sum_{k=1}^{C}exp(g_{i}^{k})}$$

Normalized probability $p_{i}$ characterizes the model’s confidence in the classification decision of $x_{i}$. The closer the $p_{i}^{k}$ value is to 1, the higher the confidence of $x_{i}$ belonging to the *k*-th class.

To optimize the model parameters from $\theta_{t}$ to $\theta_{t+1}$ at the t-th iteration, we minimized loss with a back-propagating algorithm. The model was optimized as follows:

(3) $$\theta_{t+1}=\theta_{t}-\eta *\frac{\partial L_{C}\left(y_{t}|F_{s}\left(\theta_{t},x_{t}\right)\right)}{\partial \theta_{t}}$$

where η is the learning rate, $\left(x_{t},y_{t}\right)$ is the input data of the *i*-th iteration, $\frac{\partial L_{C}\left(y_{t}|F_{s}\left(\theta_{t},x_{t}\right)\right)}{\partial \theta_{t}}$ is the partial derivative gradient of cross-entropy loss $L_{C}\left(y_{t}|F_{s}\left(\theta_{t},x_{t}\right)\right)$ to $\theta_{t}$.

Independent training in each cycle lasts for a $T_{in}$ epoch. As the loss of $L_{C}$ decreases, the student model fits the data distribution more accurately, and the more likely the sample is to be mapped to the correct classification. In the independent-training phase, student models do not communicate with each other, and they are more likely to fall into different local optimums. Student models have inconsistent information that can produce more complementary information.

### 3.3. Information Aggregation

We used soft labels as the medium of information. Soft labels are a softer normalized probability value that is more gradual and emphasizes the relationship between samples and classes. The soft-label calculation method is as follows:

(4) $${\tilde{p}}_{i}^{k}=\frac{exp(g_{i}^{k}/t)}{\sum_{k=1}^{C}exp(g_{i}^{k}/t)}$$

Parameter *t* is the temperature parameter that is used to increase the degree of relaxation of the soft label and emphasis on the secondary category. However, a too-large *t* causes confusion in the category, and we chose t=3 in our experiment.The soft-label aggregation corresponding to all inputs X is recorded as $\tilde{S},\tilde{S}=\left\{{\tilde{p}}_{0},{\tilde{p}}_{1},\ldots ,{\tilde{p}}_{N-1}\right\}$.

At the end of the independent-training period, student model $F_{si}$ calculates its own soft labels ${\tilde{S}}_{i}$ generated for input X, obtains a new data relationship $\left(X,{\tilde{S}}_{i}\right)$, and then uploads ${\tilde{S}}_{i}$ to the server.

Using a soft label to calculate cross-entropy loss:

(5) $$L_{C}\left(Y|\tilde{S}\right)=-\frac{1}{N}\sum_{i=0}^{N-1}\sum_{k=0}^{C-1}y_{i}^{k}*log{\tilde{S}}_{i}^{k}$$

Loss function $L_{C}\left(Y|\tilde{S}\right)$ is convexly optimized for soft-label value $\tilde{S}$. For the nature of convex optimization, the weighted combination of any two students’ cross-entropy loss has the following characteristics:

(6) $$L_{C}\left(Y|\left(a*{\tilde{S}}_{1}+b*{\tilde{S}}_{2}\right)\right)<=a*L_{C}\left(Y|{\tilde{S}}_{1}\right)+b*L_{C}\left(Y|{\tilde{S}}_{2}\right)$$

Simple promotion to any number of student models:

(7) $$L_{C}\left(Y|\sum_{i=0}^{M}a_{i}*{\tilde{S}}_{i}\right)<=\sum a_{i}*L_{C}\left(Y|{\tilde{S}}_{i}\right)$$

We used the weighted average approach to aggregate student information. The weighted average method is equally simple compared to the average method, but the weights can be set based on model performance to reduce the impact of interference information. Using the weighted average method can ensure that the overall cross-entropy loss of student models reducing (according to Equation (7) shows), the information of the student is effectively integrated to reduce overfitting. The students’ score weighted average process is as follows:

(8) $${\hat{S}}_{e}=\sum_{i=0}^{M}a_{i}*{\tilde{S}}_{i}$$

In the formula, ${\tilde{S}}_{i}$ are the soft labels of *i*-th students, *M* is the student number, $a_{i}$ is the weight, ${\hat{S}}_{e}$ are the aggregated soft labels. Weighted value $a_{i}$ of the student’s soft labels is determined by the accuracy: $a_{i}=\frac{v_{i}}{\sum_{k=1}^{M}v_{i}^{k}}$. Where *M* is the number of student models and $v_{i}$ is the model accuracy of student model $F_{si}$.

### 3.4. Distillation Fusion

In the distillation-fusion phase, aggregated soft labels (as teacher knowledge) ${\hat{S}}_{e}$ are used to guide student training. Minimizing the Kullback–Leibler divergence of ${\hat{S}}_{e}$ and $widetildeS_{i}$ to urge students to learn from the teacher. Kullback–Leibler divergence describes the discrepancy between students’ soft-label distribution $widetildeS_{i}$ and the teacher’s distribution ${\hat{S}}_{e}$. The Kullback–Leibler divergence-loss calculation formula of the *i*-th student is as follows:

(9) $$L_{KL}\left({\hat{S}}_{i}|{\tilde{S}}_{e}\right)=-\frac{1}{N}\sum_{\left({\tilde{p}}_{i}^{k},{\hat{p}}_{i}^{k}\right)\in \left({\tilde{S}}_{e},{\hat{S}}_{i}\right)}{\tilde{p}}_{i}^{k}*log\frac{{\tilde{p}}_{i}^{k}}{{\hat{p}}_{i}^{k}}$$

${\tilde{p}}_{i}^{k}$ is the soft-label probability of ${\tilde{S}}_{e}$ for input sample $x_{i}$ on the *k*-th class, and ${\hat{p}}_{i}^{k}$ is the soft-label probability for the $x_{i}$ in the *k*-th class on soft-label ${\hat{S}}_{i}$ of the *i*-th student. ${\tilde{S}}_{e}$ is the teacher model information that is sent to the students by the server in the previous step. ${\tilde{S}}_{e}$ is fixed in the distillation-fusion stage, and ${\hat{S}}_{i}$ is the soft-label output of the student model, updated with the update of the model parameters.

Kullback-Leibler divergence combines with standard cross-entropy loss $L_{C}$ to maintain the target of the ground-truth label value. We used a weighted approach to balance the proportion of Kullback-Leibler divergence loss and cross-entropy loss. The loss function of the *i*-th student in the distillation-fusion phase is as follows:

(10) $$L_{i}=\alpha *L_{C}^{i}+\beta *L_{KL}^{i}$$

The weights of cross-entropy loss and Kullback-Leibler divergence loss in the distillation-fusion process are α and β. We gradually changed them other than using constant values, which we call “gradual knowledge transfer”. Specifically, gradually reducing the weight of cross entropy while gradually increasing Kullback–Leibler divergence to achieve smooth loss transition in distillation.

(11) $$\alpha =1-\frac{r}{T_{d}}\;\beta =1+\frac{r}{T_{d}}$$

Here, *r* is the epoch number of the distillation, and *r* is set to 0 at the start of each training cycle. The work of distillation in generations [42] shows that gradual knowledge transfer is more effective. We note that teachers should guide students step by step. Our approach is to gradually enhance the guidance role of the teacher in each distillation cycle, which has been experimentally proven to be simple and effective. Gradually reducing the proportion of standard cross entropy during the distillation process is conducive to the smooth transition of the loss function, preventing large changes in the gradient value from causing the network to collapse. At the same time, gradually increasing Kullback–Leibler divergence loss and strengthening the guidance role of the teacher are conducive to students’ better learning.

To optimize model form $\theta_{t}$ to $\theta_{t+1}$ at the t-th iteration in distillation process, operations are a similar form of independent training.

(12) $$\theta_{t+1}=\theta_{t}-\eta *\frac{\partial L_{i}}{\partial \theta_{t}}$$

where η is the learning rate, $\left(x_{t},y_{t}\right)$ is the input data of the *i*-th distillation iteration, and $\frac{\partial L_{i}}{\partial \theta_{t}}$ is the partial derivative gradient of distillation loss $L_{i}$ to $\theta_{t}$.

The student model performs $T_{d}$ epoch distillation training in a training cycle. After the student model has trained the model through $T_{in}$ rounds of independent training and $T_{d}$ rounds of distillation training in a cycle, the model continues as the starting point for next cycle.

The deployment and training processes of the model are shown in Algorithm 1. Unlike general distillation methods, independent training and distillation training are included in one training cycle, while the aggregated information representing the teacher is updated in each new cycle. Student models participating in the training can have different network structures, but all students follow the same training process. End training until all student models are in a state of convergence. It is not allowed to exit early, so as to avoid a reduction in the overall amount of information in the model. After training is completed, select the best model among students for deployment. A collection of multiple student networks may also be chosen under conditions of sufficient memory resources and computational resources. 

**Algorithm 1:** Training process for student $F_{s}$

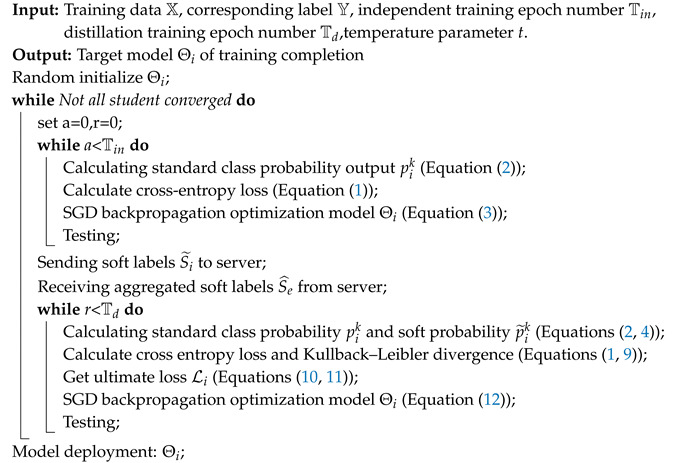



## 4. Experiments

### 4.1. Experiment Settings

**Datasets and Evaluation:** We used three widely multiclass classification benchmarks: CIFAR10, CIFAR100 [43], and ImageNet [12]. For performance metrics, we adopted the common top-n (n=1) classification accuracy.

**Neural Networks:** We used seven networks in our experiments, AlexNet [44], VGG19 [45], ResNet18, ResNet50, ResNet110 [3], SqueezeNet1-1 [18], and DenseNet100 [46].

**Implementation Details:** We used the PyTorch framework and python gRPC (communication uses) to conduct all the following experiments. The student in the experiment was configured with an Nvidia 1080Ti graphics card, and the server could use the host of the CPU processor. We followed the training strategy and first initialized learning rate 0.1, then dropped the rate from 0.1 to 0.01 halfway (50%) through training, and to 0.001 at 75%. For the hyperparameters involved in the experiment, we used $T_{in}=10,T_{d}=30$ in the CIFAR experiment, while $T_{in}=5,T_{d}=10$ in ImageNet.

### 4.2. Comparison with Vanilla Independent Learning

**Experiment Results on CIFAR**Table 1 compares the top-1 accuracy performance of varying-capacity state-of-the-art network models trained by independent conventional training and our collaborative online-distillation learning algorithms on CIFAR10/CIFAR100. From Table 1, we can make the following observations: (1) All the different networks benefit from our collaborative online-distillation learning algorithm, particularly when small models collaboratively learn with large-capacity models. Top-1 accuracy improved by 5.94% (49.79–43.85) for AlexNet when training together with VGG. This suggests a generic superiority of our online knowledge distillation across different architectures. (2) Performance gains on classification tasks with more classes (CIFAR100 VS CIFAR10) were higher for all networks. This is reasonable because richer interclass knowledge is transferred across individual architectures in an online manner to facilitate model optimization, indicating the favorable scalability of our method in solving large classification problems. (3) All of the large-capacity models benefit from joint training with small networks. Especially when ResNet collaboratively trained with VGG, top-1 accuracy improved by 3.73% (77.75–74.02).

**Experiment Results on ImageNet** We tested the large-scale ImageNet with three different networks: ResNet-18, ResNer-50, and Squeeze1-1; results are shown in Table 2. Overall, we observed a similar performance comparison with these networks as on CIFAR10/CIFAR100. This indicates the superiority of our method when testing on large-scale image-classification settings. ImageNet is large, containing more than 1000 types of image datasets. On ImageNet, we verified that this method is effective for complex tasks with very large datasets.

### 4.3. Comparison with Conventional Distillation Methods

**Figure 4:**
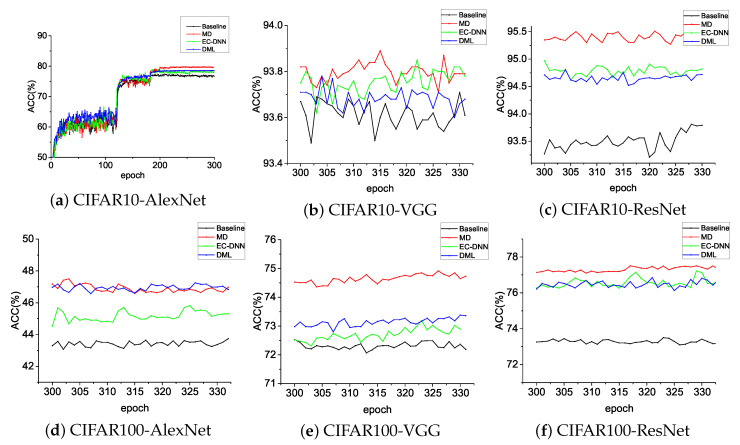
Classification accuracy compared to state-of-the-art online distillation. Baseline is the node-training-alone method; EC-DNN, Ensemble-Compression method; DML, Deep Mutual Learning method; MD, our method.

DML and Ensemble-Compression (EC-DNN) are the two most advanced distillation methods. In DML, we use two models for mutual distilling, while the EC-DNN method uses ensemble compression to improve model performance. This shows that our operation is effective: independent training was added to the process of model distillation, which increased the model difference with gradually incremental distillation weights to fuse polymerization information in distillation. Our approach still has tremendous advantages compared with state-of-the-art distillation methods, DML [25] and EC-DNN [47]. Table 3 shows that our method, MD, is almost always higher than DML and EC-DNN on CIFAR10/CIFAR100 on three typical networks: AlexNet, VGG, and ResNet. In Figure 4, wee see that MD continuously achieved higher Top-1 accuracy.

### 4.4. Ablation Study

**Figure 5:**
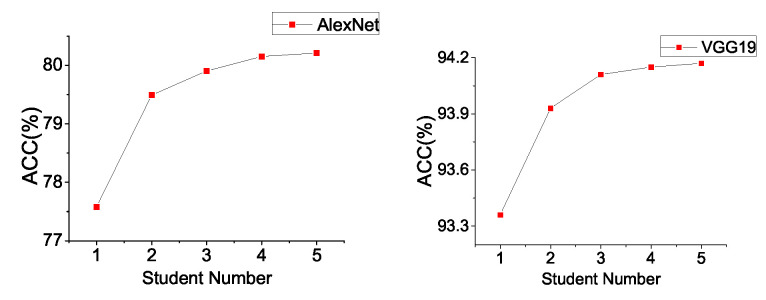
Effect on student number on CIFAR10.

**Effect on Student Number.** Cluster learning enhances the effects of model aggregation. Intuitively, different students have individual prediction distributions on the same samples. In the process of aggregating the distribution of students, the more students involved, the smaller the common error. The experiment results of AlexNet and VGG19 on CIFAR10 in Figure 5 show that the more nodes that participate in training, the better the training effect is. However, we also noticed that the bigger the network number is, the slower the growth of model accuracy.

**Analysis of Experimental Parameters.** We explored the effects of important parameters in our method, including temperature parameter *t*, the number of independent training epochs $T_{in}$ and distillation epochs $T_{d}$. On the CIFAR10 data set, we use two nodes for cooperatively distillation and setting $T_{in}=10,T_{d}=30$ to explore the influence of temperature parameters *t*. The results are shown in Table 4. We have the following conclusions:

1. The temperature parameters *t* have less impact for the same structures (AlexNet + AlexNet) compared to different structures (AlexNet + VGG19). We compared the logits statistics of AlexNet and VGG19 after 50 epochs training on CIFAR10. For AlexNet, the maximum, minimum and variance of logits are (24.41, −24.2, 8.15) while (20.5, −11.3, 11.42) for VGG19. It is more important to use an appropriate temperature parameter to unify the classification score scale for networks with different structures.

2. In the experiments of (AlexNet + VGG19), the best results (AlexNet: 80.23, VGG: 93.81) are obtained with t=3. The classification accuracy decreases when t>5, which show that a bigger *t* obscures the major class probability. The gradient explosion occurs when t=0.5 or 1. A smaller *t* leading to the loss of secondary class information, and the values of soft labels are close to discrete 0 and 1 which causes the difficulty in model optimization with Kullback-Leibler divergence.

Setting temperature parameter t=3, the relationship between the number of independent training epochs $T_{in}$ and distillation training epochs $T_{d}$ is shown in Table 5:

We observed that the best results were obtained when $T_{in}=10,Td=30$. Fixed $T_{in}=10$, with the increase of the number of distillation epochs $T_{d}$, the accuracy increases gradually and stop increasing after $T_{d}=30$. Such changes show that a short distillation process cannot adequately transfer teacher’s knowledge to the student networks, resulting in the loss of information.

**Gradual Knowledge Transfer.** An article on knowledge distillation in generations [42] suggests that teachers who are tolerant usually educate better teachers and produce better results by gradually increasing the constraints of the teacher. We used a multicycle training process in which gradual strict KL constraints are used in each cycle of the distillation phase. Validated on CIFAR10 and CIFAR100, our method compares the use of generally incremental distillation weights ((gMD), $T_{in}=10,T_{d}=30,$ $\alpha =1-r/T_{d},\beta =1+r/T_{d}$) with fixed distillation weights (fMD), $T_{in}=10,T_{d}=30,\alpha =1,\beta =2$). Experiment results are shown in Table 6.

On both the CIFAR10 and CIFAR100 datasets, AlexNet and VGG19 were more accurate at using gMD than using fMD. Increasing teacher constraints enhances the distillation effect.

## 5. Conclusions and Future Work

In this paper, we proposed a collaborative framework using ensemble and distillation mechanisms, which helps participating nodes learn to integrate the network information of other nodes during training to achieve better performance. Our approach uses soft labels to pass model information and make it compatible with different networks and training methods. The use of progressively increasing distillation and adding independent-training phase constraints enhances the effectiveness of the method. The experiment verified that our method surpassed the best distillation method of the time. Our method has wide applicability in classification tasks. In the hopes of helping other researchers, competition members, and industries to train better networks, our future work will explore the method of intermediate distillation and the effectiveness of distillation in other scenarios. 

## Figures and Tables

**Table 1:** Top-1 accuracy (%) on the CIFAR10 and CIFAR100 datasets. I represents independent training and MD represents our method. m1 and m2 are the abbreviations of Model1 and Model2.

**CIFAR10 Results**					
**Model1**	**Model2**	**I (m1)**	**I (m2)**	**MD (m1)**	**MD (m2)**
VGG	ResNet	93.36	93.84	**94.27**	**95.6**
DenseNet	ResNet	95.35	93.84	**95.72**	**95.48**
AlexNet	VGG	77.58	93.36	**80.23**	**93.81**
ResNet	ResNet	93.84	93.84	**95.72**	**95.75**
DenseNet	DenseNet	95.35	95.35	**95.62**	**95.76**
VGG	VGG	93.36	93.36	**94.10**	**94.33**
**CIFAR100 Results**					
**Model1**	**Model2**	**I (m1)**	**I (m2)**	**MD (m1)**	**MD (m2)**
VGG	ResNet	72.57	74.02	**74.85**	**77.75**
DenseNet	ResNet	77.57	74.02	**78.57**	**79.09**
AlexNet	VGG	43.85	72.57	**49.79**	**74.08**
ResNet	ResNet	74.02	74.02	**77.81**	**77.47**
DenseNet	DenseNet	77.57	77.57	**78.55**	**78.85**
VGG	VGG	72.57	72.57	**75.43**	**75.45**

**Table 2:** Experiment test accuracy (%) on ImageNet.

Model1	Model2	I (m1)	I (m2)	MD (m1)	MD (m2)
ResNet18	ResNet18	69.76	69.76	**70.53**	**70.44**
ResNet18	ResNet50	69.76	76.15	**70.65**	**76.38**
ResNet18	SqueezNet	69.76	58.1	**70.11**	**58.98**

**Table 3:** Comparison with state-of-the-art online-distillation methods. Red/Blue: Best and second-best results.

Network	AlexNet		ResNet-110	
Datasets	CIFAR10	CIFAR100	CIFAR10	CIFAR100
Baseline	77.58	43.85	93.84	74.02
DML	78.6	**47.32**	94.81	76.92
EC-DNN	**78.67**	46.08	**94.97**	**77.38**
MD	**79.87**	**50.13**	**95.56**	**77.81**

**Table 4:** Setting the number of independent training epochs $T_{in}=10$ and the number of distillation epochs $T_{d}=30$, the influence of temperature parameters on accuracy (%) is studied. The same structure (AlexNet + AlexNet) and different structure (AlexNet + VGG19) are compared in the experiment.

Temperature (*t*)	0.5	1	2	3	4	5	6	9
AlexNet	NaN	79.37	79.3	**79.51**	79.3	79.43	79.35	79.42
AlexNet	NaN	79.56	79.21	79.59	79.46	**79.68**	79.28	79.48
AlexNet	NaN	NaN	79.97	80.23	80.22	**80.44**	79.79	80.17
VGG19	NaN	NaN	93.72	**93.81**	93.41	93.46	93.67	93.68

**Table 5:** Setting temperature parameter t=3, the number of independent training epochs $T_{in}=10$, change the number of distillation epochs $T_{d}$, the accuracy (%) of cooperative training of two nodes (AlexNet + AlexNet) using our method.

Distillation Epochs ($T_{d}$)	1	5	10	20	30	40
AlexNet	77.72	78.29	78.9	78.84	**79.43**	79.41
AlexNet	77.96	78.54	78.96	79.04	**79.59**	79.31

**Table 6:** Results comparison of whether to use the gradual enhancement of the teacher-constraint method. gMD, incremental distillation weights; fMD, fixed distillation weights.

Dataset	CIFAR10			CIFAR100		
Model	Baseline	fMD	gMD	Baseline	fMD	gMD
AlexNet	77.58	79.49	79.87	43.85	49.96	50.13
VGG19	93.36	93.93	94.33	72.57	75.32	75.45
